# The School-Room as a Factor in Diseases of Young Girls

**Published:** 1906-08

**Authors:** 


### The School-Room as a Factor in Diseases of Young Girls.

---

There is no disguising the fact that our system of imparting knowledge by imposing excessive intellectual labor and stimulating competitive zeal in the school-room is very largely responsible for most of the nervous disorders of the young women of today.

That sustained mental exertion is a menace to the health of girls at the age of puberty, there can be no doubt denying. Yet that is precisely the system in vogue at our institutions of learning at the present time.

While it is true that modern architecture has greatly improved the hygienic condition of the study-rooms, it is highly probable that the present rush and hurry methods of instruction are even more injurious to the physical state of our young women than was the faulty system of ventilation, until recently endured.

The worry and excitement attendant upon present-day school life is, undoubtedly, the prime cause of a governing percentage of the neurotic disturbances which are so prevalent among the women of America. In fact, it is quite within the bounds of truth to assert that many of the diseases which present themselves to the gynecologist have for their origin a nervous system rendered bankrupt by strife in our temples of education.

Mental over-strain, when enforced day after day, soon renders the nerve structure incapable of absorbing adequate nourishment from the blood stream. Ultimately, nervous vitality is almost completely exhausted and depression, gloom, laughter and mental impotence ensue.

As the taxation is extended, the condition grows worse until anemia, anorexia, insomnia, melancholia, and, perhaps, hysteria develop.

Inasmuch as it is not within the power of the physician to remedy this evil system of handling our young women, it remains for him to evolve means of attenuating, as far as possible, the injury done, and preventing the development of lasting diseases which have their origin in the shattered nervous system.

This is best accomplished by the upbuilding of the psychical and physical resources of the individual. Not by the employment of stimulants which act ephemerally upon the organism, but by encouraging functional activity to its maximum degree consistent, of course, with normality.

Obviously, this must be done by maintaining the entire digestive system at its proper standard, for it is through these channels that vital force is obtained and the wellbeing of the economy is preserved.

It is not consistent with logic to achieve this end by resort to the employment of those agents which relieve the digestive secretions of their special provinces. On the contrary, it is the very extreme of indiscretion to encourage dependency of the gastric or intestinal fluids, or to aid them in the performance of their duties beyond very circumscribed limits.

Quite the most rational course to pursue is that of extending to Nature gentle, but ample, encouragement through the administration of an agent which is capable of bringing functional activity to its highest point without entailing the necessity of prolonged drugging. It is supremely important that the drug be one that can be withdrawn without leaving the economy disqualified to maintain a normal fund of vital force.

It is here that iron is of the greatest therapeutic use. Not only does it impart to the blood stream a full measure of nutrition-conveyors in the form of hemoglobin, but it substantially increases the capacity of the tissues to absorb and utilize the nourishment placed at their disposal by the circulatory system.

Further, iron, when administered in the proper form, augments functional activity throughout the entire digestive apparatus, and, thuswise, enables the economy to secure the full benefit of the food supply. To this action of the drug is due the greatest profit to the individual resulting from its use.

The objections applicable to some forms of iron gain added importance in this particular class of cases, for the reason that the peculiarities of the disorders under consideration are such as to be greatly aggravated by an improper form of iron.

Chief among these peculiarities is constipation, which is invariably a disturbing factor. The existing constipation is easily made worse by both the carbonate and acid solutions of iron; and, in fact, these forms of the drug are notably stool-discouraging. Digestive processes are also depressed by these forms of iron, and headache frequently follows their use.

Partly because of these objections, but mainly on account of its manifold advantages, Pepto-Mangan (Gude) is given the preference over all other forms of iron, and a mass of clinical data has been brought forth to sustain this opinion. Pepto-Mangan (Gude) is of the greatest aid in the treatment of all the ill-defined disorders commonly encountered among school-girls who exhibit a tendency to anemia, nervous debility, anorexia, moroseness and mental depression.

Obviously, this general emphatic endorsement of Pepto-Mangan (Gude) by the most exacting members of the profession is based upon a critical analysis of its therapeutic advantages over the ancient forms of iron.

